# Cytotoxic Effects and Intracellular Localization of Bin Toxin from *Lysinibacillus sphaericus* in Human Liver Cancer Cell Line

**DOI:** 10.3390/toxins13040288

**Published:** 2021-04-19

**Authors:** Simab Kanwal, Shalini Abeysinghe, Monrudee Srisaisup, Panadda Boonserm

**Affiliations:** Institute of Molecular Biosciences, Mahidol University, Salaya, Phuttamonthon, Nakhon Pathom 73170, Thailand; simk.biochem@gmail.com (S.K.); shaliniabeysinghe90@gmail.com (S.A.); catta_w255@hotmail.co.th (M.S.)

**Keywords:** *Lysinibacillus sphaericus*, Bin toxin, parasporin-2 toxin, cytotoxicity, HepG2 cells, toxin internalization

## Abstract

Binary toxin (Bin toxin), BinA and BinB, produced by *Lysinibacillus sphaericus* has been used as a mosquito-control agent due to its high toxicity against the mosquito larvae. The crystal structures of Bin toxin and non-insecticidal but cytotoxic parasporin-2 toxin share some common structural features with those of the aerolysin-like toxin family, thus suggesting a common mechanism of pore formation of these toxins. Here we explored the possible cytotoxicity of Bin proteins (BinA, BinB and BinA + BinB) against Hs68 and HepG2 cell lines. The cytotoxicity of Bin proteins was evaluated using the trypan blue exclusion assay, MTT assay, morphological analysis and LDH efflux assay. The intracellular localization of Bin toxin in HepG2 cells was assessed by confocal laser scanning microscope. HepG2 cells treated with BinA and BinB (50 µg/mL) showed modified cell morphological features and reduced cell viability. Bin toxin showed no toxicity against Hs68 cells. The EC_50_ values against HepG2 at 24 h were 24 ng/mL for PS2 and 46.56 and 39.72 µg/mL for BinA and BinB, respectively. The induction of apoptosis in treated HepG2 cells was confirmed by upregulation of caspase levels. The results indicated that BinB mediates the translocation of BinA in HepG2 cells and subsequently associates with mitochondria. The study supports the possible development of Bin toxin as either an anticancer agent or a selective delivery vehicle of anticancer agents to target mitochondria of human cancer cells in the future.

## 1. Introduction

Binary toxin (Bin toxin) is produced as crystalline inclusions during the sporulation stage of *Lysinibacillus sphaericus* (hereafter *L. sphaericus*). Bin toxin contains two crystal proteins, namely BinA (Tpp1Aa2) and BinB (Tpp2Aa2) with the sizes of 42 and 51 kDa, respectively, and both of them act together to exert their toxicity against *Culex* and *Anopheles* mosquito larvae [1]. Both BinA and BinB share little amino acid sequence homology with those from other bacterial toxins (such as insecticidal toxins), but they share 25% sequence identity and 40% similarity among themselves [2]. Despite their similarities, these two proteins have distinct functions: BinB serves as a receptor-binding component, while BinA is responsible for toxicity inside the target cells. The mechanism of Bin toxin internalization was previously analyzed by using fluorescently labeled toxins. *Culex* larvae were fed with fluorescently labeled BinA and BinB, and the dissected larvae were visualized by using a confocal laser scanning microscope. When fed together, both BinA and BinB were co-localized on the cell surface and cytoplasm of *Culex* larval gut cells. However, BinA alone was found to localize only on the cell membrane, whereas BinB alone was detected both on the cell membrane and in the cytoplasm of larval gut cells. This result suggested that internalization of BinA is crucial to exert toxicity and BinB facilitates this internalization into susceptible larval gut cells through receptor-mediated endocytosis. The internalized BinA is supposed to induce autophagy or cytotoxicity that leads to cell death via apoptosis [3,4].

The three-dimensional structures of BinA and BinB protoxins and activated BinB have been solved by X-ray crystallography [5,6]. The *N*-terminal domain is globular and has been proposed to function as receptor binding, based on its structural similarities with sugar-binding proteins or lectins. The *C*-terminal domains of BinA and BinB show partial homology with aerolysin-type β pore-forming toxins (β-PFTs), including the parasporin-2 (Cry46Aa1 or Mpp46) (hereafter PS2) [5,6,7] which is produced by *Bacillus thuringiensis* (Bt) with high cytotoxicity towards some human cancer cell lines such as HepG2, MCF-7 and PC-3 cells but non-cytotoxicity to normal cell lines. When the *C*-terminal domain of BinB was compared with that of PS2, partial homology was found in PS2 (2ZTB) [7] with rmsd of 5.5 Å. The HepG2 cells intoxicated by PS2 resulted in cytochrome c leakage from mitochondria, indicating an apoptotic induction event [8]. Furthermore, caspase activation and the regulation of survival/death pathways such as AKTs have been shown to be involved in the cell death via apoptosis induction of PS2 [9,10]. Previously, it was also reported that a high concentration of *L. sphaericus* originated Bin toxin induces cytotoxicity in various human cancer cell lines and might be involved in apoptosis induction in nasopharyngeal carcinoma cells (HK-1) [11].

Given the structural and functional similarities, we hypothesize that Bin toxin may exhibit cytotoxicity against human liver cancer cells as observed in PS2. Therefore, this study aimed to investigate the anticancer activity of optimized concentrations of BinA, BinB and their combination against a human liver cancer cell line (HepG2) and a human fibroblast cell line (Hs68) as a normal cell line. The evidence for apoptotic progression was evaluated in cytotoxic cells by analyzing the caspase levels. Both BinA and BinB were found to be internalized into the cytoplasm of HepG2 cells and to associate transiently with mitochondria, supporting the possible development of Bin toxin as either an anticancer agent or a selective delivery vehicle of anticancer agents to target intracellular compartments of human cancer cells in the future.

## 2. Results and Discussion

### 2.1. Cytotoxic Effects of Protein Toxins on Cancer and Normal Cells

**Figure 1:**
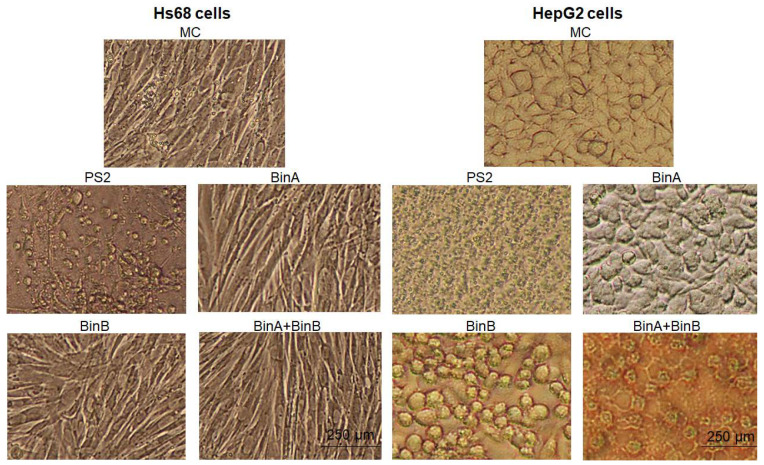
Morphology of Hs68 and HepG2 cells treated with mock control (MC; 1× PBS buffer) and protein toxins. Cells were treated with PS2 (0.5 µg/mL) and Bin proteins (50 µg/mL) for 24 h and then viewed by inverted light microscope (magnified 10×).

Among several human cancer cells, human liver cancer cell line, HepG2, was reported to be a highly susceptible cell line to PS2 [11]. Therefore, to explore the cytotoxic effects of Bin toxin on human cancer cell lines compared with PS2, HepG2 cell line was used as a model system in this study. Previously, Bin proteins (BinA, BinB and BinA + BinB) at high concentrations have been reported to inhibit the cell proliferation of A549 and HepG2 cells [11,12]. In the present study, the cytotoxic effects on human cells were investigated at comparatively lower concentrations of protein toxins. The morphological alterations induced by protein toxins during 24 h incubation in Hs68 and HepG2 cell lines were observed by inverted light microscopy as presented in Figure 1. No cytopathic change was observed in Hs68 cells upon Bin toxin treatment when compared with mock control (MC)-treated cells, whereas PS2 (0.5 µg/mL), which is known to cause cell killing via creating pores in the plasma membrane of cells [13], caused cell blebbing and clustering in Hs68 cells. On the other hand, complete cell necrosis and lysis were observed in HepG2 cells treated with PS2. The treatment with BinA (50 µg/mL) seemed to inhibit the proliferation of HepG2 cells when compared with MC-treated cells, whereas both BinB (50 µg/mL) and BinA + BinB (1:1) treatments altered the cell morphology by forming cell clumping and shrinkage along with an antiproliferative effect on HepG2 cells. Mesh formation was also observed in HepG2 cells treated with BinA + BinB. These results suggest that Bin and PS2 exert different cytotoxic patterns towards the target cells.

**Figure 2:**
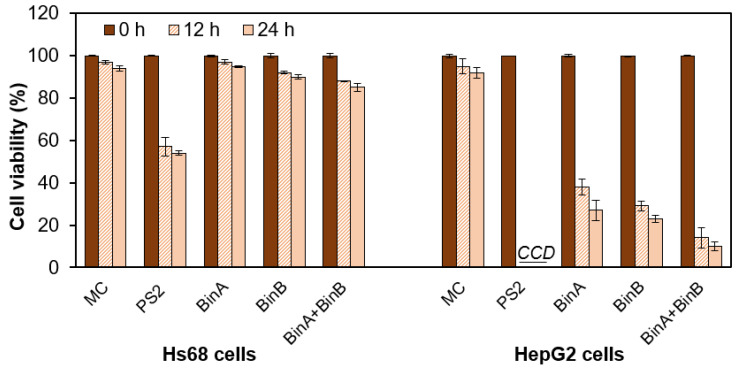
Assessment of one-dose cytotoxic effect of proteins on human fibroblast and cancer cell viability by trypan blue assay. Cells (Hs68 and HepG2) were treated with 0.5 and 50 µg/mL of PS2 and Bin proteins, respectively, at indicated time intervals prior to the assay. In each assay, 1× PBS was used as a mock control (MC). Data correspond to mean values ± standard deviation from at least three different experiments. CCD = complete cell death.

Activated Bin toxin from *L. sphaericus* significantly affected the HepG2 cell viability as examined from one-dose trypan blue exclusion assay for 12 and 24 h treatment (Figure 2). Up to 12 h after inoculation, BinB caused a decrease in cell viability resulting in ~29% cell survival rate in HepG2 cells, and BinA caused ~38% cell survival rate. Notably, BinA + BinB (1:1) treatment decreased the cell viability to ~14%. In contrast, the viability of Hs68 cells remained completely unaffected upon BinA treatment, whereas BinB and BinA + BinB had minor cytotoxic effects (~5–9% when compared to MC-treated Hs68 cells). Cells showed >94% viability when treated with MC. When the incubation period was extended to 24 h, the viability of HepG2 cells further decreased to ~11%, 6% and 4% for BinA, BinB and BinA + BinB treatments, respectively. However, the viability of Hs68 cells was mildly affected by extending the incubation period to 24 h with protein toxins. The results obtained from the cytotoxic effects of Bin proteins on cancer cells were found concomitant with the cell morphological changes induced by the toxin and also confirm the non-cytotoxicity of Bin proteins on normal cells. As compared to the cytotoxicity induced by Bin toxin, HepG2 cells were found to be more sensitive to PS2, which caused complete cell death. This agrees with the previous studies on HepG2 cells, showing the involvement of efficient cytocidal action of PS2 protein via pore formation in the membrane that leads to organelle fragmentation, cell swelling and ultimately cell lysis [9,10]. However, in the present study, it was observed that PS2 caused significant cell death (~46% reduction in cell survival rate) in Hs68 cells, which was quite contrary to various previous reports that suggested that normal cells such as normal lymphocytes, Vero (monkey kidney normal cells), normal mouse splenocytes and thymocytes show either no or minimal sensitivity toward parasporin proteins [14,15].

**Figure 3:**
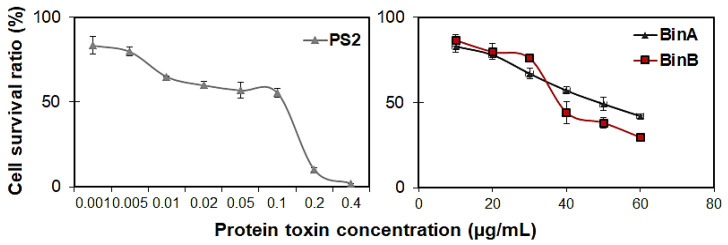
Concentration–response curves for the protein toxins PS2, BinA and BinB against HepG2 cell line. The cell survival rate was evaluated 24 h after inoculation with increasing concentrations of the protein toxins using the MTT assay. The data are the means ± SDs of triplicate analyses.

The results of the concentration–response study conducted by MTT assay in HepG2 cells indicated that the cell survival rate is significantly affected by protein toxins in a dose-dependent manner (Figure 3). The EC_50_ value for PS2 protein deduced from concentration–response analyses at 24 h after inoculation was 24 ng/mL, whereas, BinA and BinB had EC_50_ values of 46.56 and 39.72 µg/mL against HepG2 cells, respectively (Table 1).

The decrease in the cytotoxic effect on HepG2 cells caused by the combination of Bin subunits in comparison to individual Bin toxin subunits observed in the present study was in concordance with a previous report by Chankamngoen et al. [11]. In fact, Surya et al. [16] indicated that change in conformational state of Bin toxin monomeric subunits to heterodimeric form occurs when present together in an equimolar ratio in an aqueous solution. The formation of heterodimers in BinA + BinB might affect the interaction of Bin toxin with membrane or with the targeted receptor in susceptible cancer cell lines, resulting in the difference in cytotoxic effects. On the other hand, despite the structural similarities of Bin toxin and PS2, the results from our study indicate that the cytopathological effects of Bin toxin on HepG2 are possibly caused in a manner distinct from that of PS2. To further test our hypothesis, the effects of protein toxins on plasma membrane integrity and apoptotic characteristics of human cells were assessed.

### 2.2. Effect of Protein Toxins on Plasma Membrane Permeability and Caspase Activation in Human Cells

**Figure 4:**
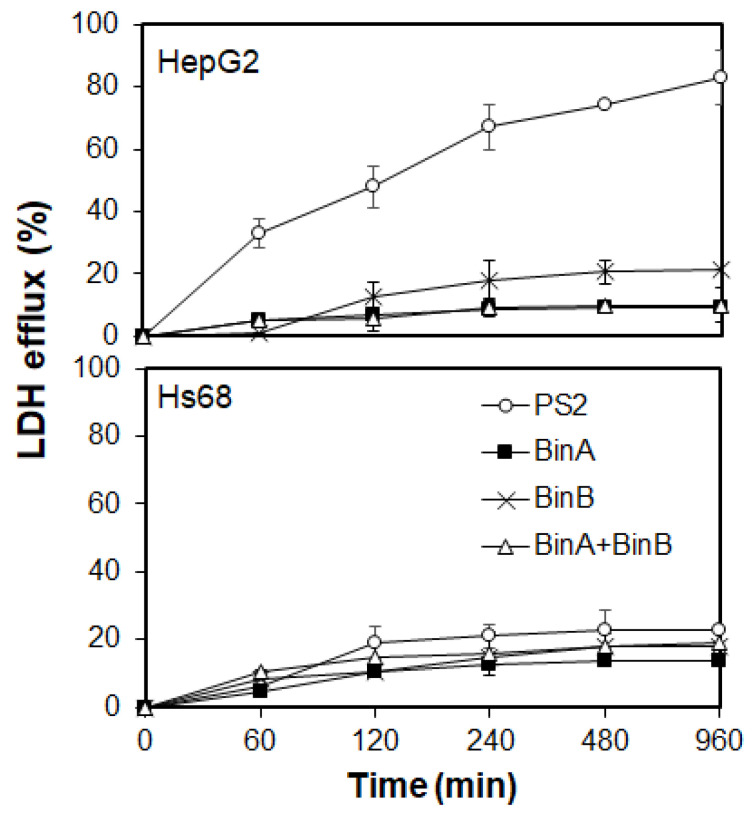
The effect of protein toxins on plasma membrane permeability of Hs68 and HepG2 cells assessed by LDH efflux assay. HepG2 cells were incubated with PS2 and Bin toxin at the EC_50_, whereas Hs68 cells were tested at 0.5 and 50 µg/mL of PS2 and Bin proteins, respectively, for the indicated time, and the LDH activity in the medium was assessed by LDH-Cytotoxicity Assay Kit II (Abcam). LDH efflux (%) was expressed as the ratio to the untreated control cells and maximum release of LDH from the control cells by the addition of a lysis solution included in the kit. The data represent three independent experiments, and representative data were plotted with standard deviations.

Cytocidal actions induced by various types of β-PFTs involve different mechanisms such as apoptotic cell death or pore formation in the plasma membrane of cells [13,14]. Studies emphasize that cytocidal actions induced by Bt-derived Cry toxins and Bin toxin in target cells of insect larvae are caused by receptor-mediated membrane interactions and cytoplasmic leakage leading to ultimate cell lysis [15,17], a mechanism similar to the cytocidal activity of β-PFTs. However, it is not clear whether the Bin-toxin-mediated cytocidal action on HepG2 cells is elicited by cell lysis via membrane disruption or cellular internalization resulting in intracellular cytotoxicity or apoptotic cell death. To confirm the mode of cell death, additional characterization of intracellular events in HepG2 cells treated with Bin proteins was completed to investigate the possible involvement of apoptosis. The effect of protein toxins on plasma membrane permeability of Hs68 and HepG2 cells was investigated by monitoring the LDH leakage from the cytoplasm of cells. LDH present in all cell types is instantly released into the cell culture medium upon plasma membrane damage, and its extracellular levels could be determined colorimetrically. The rate of LDH efflux accounts for the population of damaged cells and hence determines the cytotoxicity levels caused by disruption of plasma membrane permeability. In the present study, both cell lines were treated with Bin proteins and PS2 as described in Section 3.3 and incubated for 1 to 16 h at 37 °C prior to performing LDH assay. The LDH efflux increased in PS2- and BinB-treated HepG2 cells with the increase in incubation time, as shown in Figure 4. The LDH leakage from BinB-treated HepG2 cells was slightly higher than that from cells treated with BinA and BinA + BinB, although the overall LDH leakage was not too high in both of the Bin-toxin-treated HepG2 and Hs68 cells, suggesting that Bin toxin does not induce noticeable damage to the membrane permeability. A significant escalation in LDH efflux in PS2-treated HepG2 cells was detected within 60 min, depicting substantial damage caused by PS2 to the plasma membrane permeability leading to cell lysis and death corresponding to the cytotoxic effects explained earlier in this study (Figure 1 and Figure 2). Our results are supported by the previous study of Kitada et al. [13] on the cytocidal effects of PS2 on HepG2 cells, elucidating that this toxin causes rapid membrane depolarization in HepG2 cells following protein efflux from the cells. However, PS2 did not seem to cause significant damage to the plasma membrane of Hs68 cells, as apparent from the lower LDH efflux. These findings suggest that Bin toxin might not cause pore formation in the plasma membrane of susceptible cancer cells as compared with PS2 that causes noticeable damage to the plasma membrane permeability, manifesting a typical characteristic of the β-PFT family.

**Figure 5:**
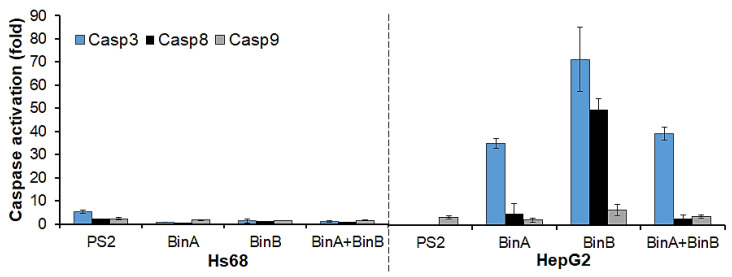
Assessment of caspase 3, 8 and 9 levels in Hs68 and HepG2 cells after exposure to protein toxins for 24 h. HepG2 cells were treated with PS2 and Bin toxin at the EC_50_, whereas Hs68 cells were treated at 0.5 and 50 µg/mL of PS2 and Bin proteins, respectively. The levels of caspases were measured using Caspase 3, 8 and 9 Multiplex Activity Assay at room temperature. Readings obtained from treated cells were compared with measurements from control cells (untreated cells) using fold increases. Each experiment was performed in duplicate.

To further test the possibility of apoptosis induction by protein toxins in Hs68 and HepG2 cells, the levels of apoptotic mediators (caspases 3, 8 and 9) were determined using Caspase 3, 8 and 9 Multiplex Activity Assay. The activity levels (Figure 5) of initiator Casp8 and executioner Casp3 were highly elevated in BinA- and BinB-treated HepG2 cells. The Casp3 and 8 activities were increased by ~35- and 5-fold in BinA-treated cells, respectively. Respective increments of ~71- and 50-fold in Casp3 and 8 activity levels were observed in BinB-treated cells. The activity level of Casp9 was increased up to ~6.5-fold in response to BinB treatment. On the other hand, BinA + BinB treatment resulted in an increase in Casp3 activity up to ~39-fold, but little increase in activity levels of Casp8 (about 3-fold) and Casp9 (about 4-fold) was observed. The PS2 treatment in HepG2 cells tends to enhance the activity of Casp9 by ~3.4-fold, whereas activities of Casp3 and 8 were not detected. These results are in line with the previous study by Brasseur et al. [9] in which the presence of cleaved Casp9 via Western blot analysis in PS2-treated HepG2 cells was reported but Casp8 cleavage/activation was not observed. It was also noticed that no caspase activity could be seen in HepG2 cells treated for 24 h with PS2 at higher concentration (0.5 µg/mL) (data not shown), as the cells had already undergone necrosis and complete lysis prior to induction in caspase activity owing to significant plasma membrane damage caused by PS2. Even though the cell damage had apparently proceeded, apoptotic processes do occur in an elusive manner. It was also mentioned previously [13] that caspase activation is not observed in HepG2 cells treated with PS2 at higher doses. On the other hand, Bin proteins did not seem to alter the plasma membrane integrity of HepG2 cells as much as PS2 (Figure 4) and instead reduced the cell viability by causing the intracellular apoptotic events after translocating inside the cells, which is in concordance with the decline in cell survival rate observed in the cytotoxicity assay (Figure 2). In the case of Hs68 cells treated with Bin proteins, no significant increase in caspase activity could be measured by the caspase assay. However, in this cell line, PS2 treatment induced the Casp3 activity up to ~5-fold. The cytocidal action of PS2 on Hs68 cells was never studied before, but other normal tissue cell lines that were studied were not found susceptible to PS2 [13,14]. However, our results seem to contradict the previous studies [9,13,14] stating that PS2 has no or little cytotoxic effect on normal tissue cells; in the present study, a cell death rate of approximately 46% was accompanied by marked cytopathic changes (Figure 1 and Figure 2), and Casp3 activation (Figure 5) was noticed in Hs68 cells in response to 0.5 µg/mL of PS2.

In caspase-dependent apoptosis (programmed cell death), Casp3, the effector caspase, requires initiator caspases from either the intrinsic (Casp9) or extrinsic (Casp8) pathway for its activation [18]. Our results indicated that activity levels of Casp3 were very high in BinA- and BinB-treated cancer cells and corresponded to the activity levels of Casp8, reinforcing the idea of the induction of the extrinsic apoptosis pathway by individual Bin toxin subunits. Moreover, the BinA + BinB treatment increased the activities of Casp3 and Casp9 (which had activity slightly higher than Casp8) in HepG2 cells, indicating the induction of the mitochondrial apoptosis pathway [19]. The association of Bin toxin with HepG2 mitochondria is discussed in the next section.

### 2.3. Intracellular Localization and Mitochondrial Association of Bin Toxin in HepG2 Cells

**Figure 6:**
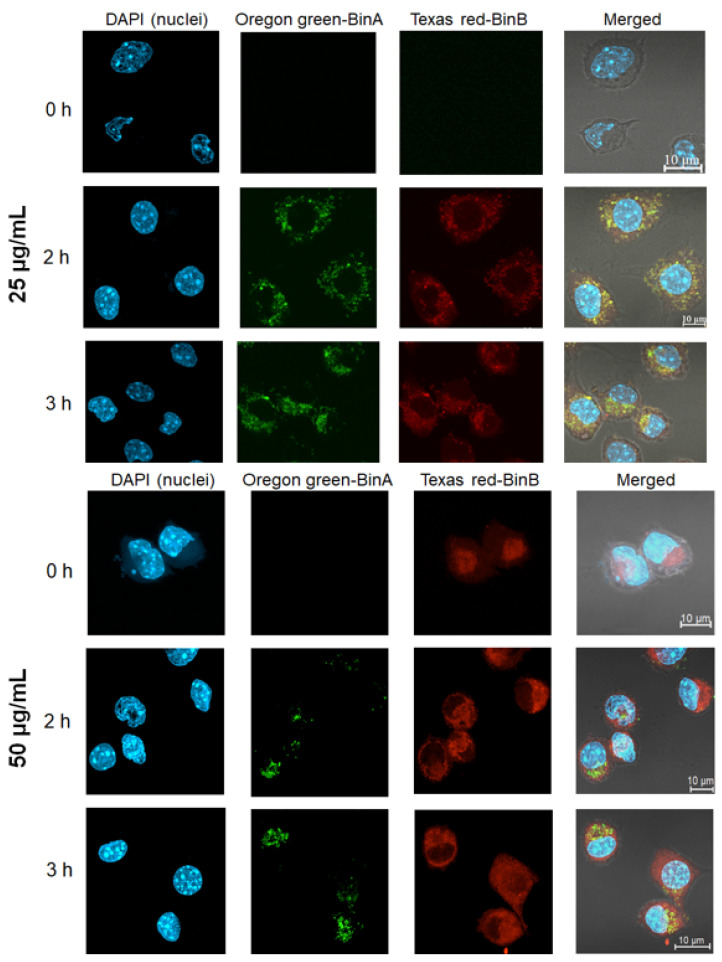
Fluorescence confocal microscopy of HepG2 cells treated with Oregon Green labeled BinA/Texas Red labeled BinB mixture. HepG2 cells were incubated with 25 and 50 μg/mL of Oregon Green labeled BinA/Texas Red labeled BinB mixture for 2 and 3 h to observe the internalization pattern in the cells. The nuclei were stained with DAPI. Three-dimensional views of Oregon Green labeled BinA/Texas Red labeled BinB treated cells were constructed from the selected depth two-dimensional images. Approximately 30 cells for each condition were analyzed. The localization of Oregon Green labeled BinA and Texas Red labeled BinB was observed using laser scanning confocal microscope, and images were processed from a three-dimensional view using Zen blue software.

To gain an in-depth understanding of the cytotoxic activity of Bin in the HepG2 cell line, BinA and BinB proteins fluorescently labeled with Oregon Green and Texas Red, respectively, were tracked for their internalization and co-localization patterns in the cells. The analysis of co-localization pattern was conducted with a combination of Oregon Green labeled BinA and Texas Red labeled BinB (1:1) at 25 µg/mL and 50 µg/mL, and the efficiency of labeling was calculated by Beer–Lambert law. The degree of labeling for Oregon Green labeled BinA and Texas Red labeled BinB was calculated as 1.42 and 1.59, respectively. Both Texas Red and Oregon Green succinimidyl ester dyes, also known as amine-reactive dyes, bind with the amine groups of the proteins. The biological activity of Bin toxin with a similar fluorescent labeling was previously analyzed via in vivo biological assay by feeding Bin toxin to *Culex quinquefasciatus* larvae that showed comparable LC_50_ values of fluorescently-labeled and unlabeled Bin toxin, indicating no functional alteration upon fluorescent labeling [3]. The intracellular co-localization of 25 µg/mL of Oregon Green labeled BinA/Texas Red labeled BinB was observed as yellow fluorescence after 2 h of toxin treatment, as shown in Figure 6. It was found that Texas Red labeled BinB was quickly internalized in the HepG2 cytoplasm; in comparison, Oregon Green labeled BinA was mostly localized in the cell membrane (data not shown). Similar internalization of Bin toxin was previously observed in susceptible mosquito gut cells, where BinA was detected only on the cell membrane but could not be detected inside the cytoplasm of mosquito gut cells, whereas BinB was detected inside the cytoplasm of susceptible mosquito midgut cells, suggesting that BinB alone can enter into mosquito larvae gut cells [3]. The toxin internalization signals were intensified with the increase in the concentration to 50 µg/mL and the increase in the incubation time up to 3 h. It is not clear yet whether the BinA/BinB complex was transiently formed during membrane translocation, as BinA and BinB free molecules were also detected after localization in HepG2 cell cytoplasm. It might be possible that the BinA/BinB complex transiently formed prior to internalization undergoes dissociation after intracellular localization.

**Figure 7:**
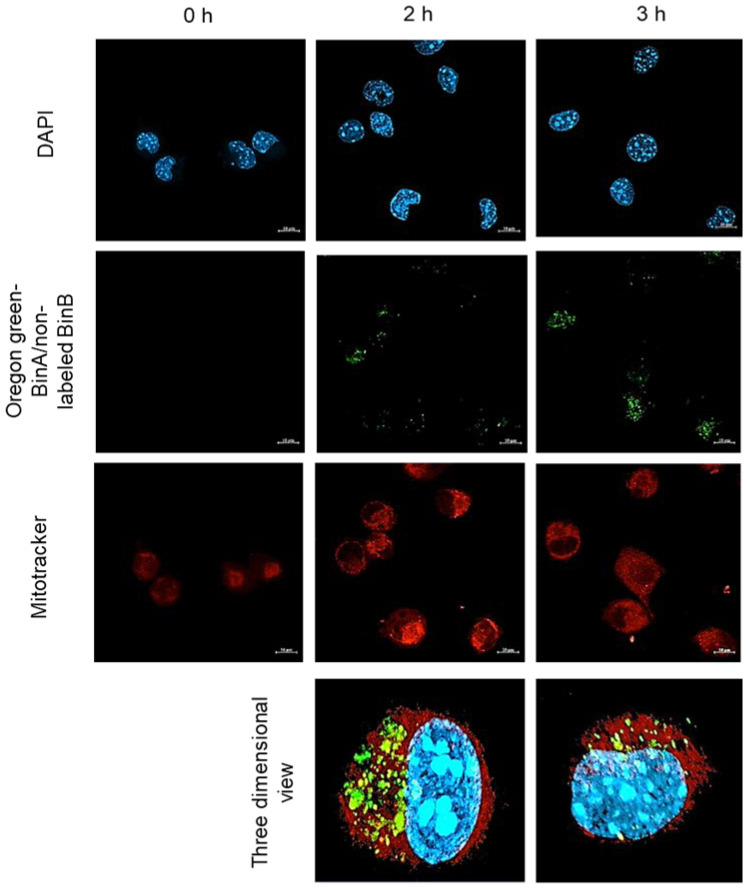
Bin toxin association with mitochondria. HepG2 cells were treated with Oregon Green labeled BinA and unlabeled BinB mixture in 1:1 ratio at 50 µg/mL for 0, 2 and 3 h. The mitochondria and nuclei were stained with Mitotracker CMXRos and DAPI (4′,6-diamidino-2-phenylindole) respectively. Two-dimensional images were constructed from a three-dimensional view of the treatment detected with confocal microscopy.

The association of internalized Bin toxin with mitochondria of HepG2 cells was investigated by treating the cells with a 1:1 combination of 50 µg/mL of Oregon Green labeled BinA and non-labeled BinB. The mitochondria were labeled with Mitotracker Red to detect the co-localized toxin in yellow fluorescent signal. The confocal images showed that the mitochondria were stained with red signal that was distributed throughout the cytoplasm of HepG2 cells (Figure 7). The fluorescent signal of Oregon Green labeled BinA/nonlabeled BinB was detected after 2 and 3 h of toxin treatment, and the signal was slightly co-localized with mitochondria giving a yellow fluorescent signal. Results revealed that BinB toxin was internalized and localized in the cytoplasm of HepG2 cells after 2 h of incubation as the signal could be seen throughout the cytoplasm. However, the co-localization of BinA with mitochondria was not as specific and strong as that of BinB as analyzed from Pearson’s coefficient values (data not shown). It was reported previously that Bin toxin induces cell vacuolization and mitochondrial swelling in *Culex* larval gut cells [20,21,22]. Although BinB seems to facilitate the internalization of BinA, the BinA/BinB complex might undergo dissociation after internalization in the cytoplasm of HepG2 cells, resulting in a lower association of BinA with mitochondria.

Considering the lower cytotoxicity of Bin toxin than that of PS2, the structural diversities may contribute to their different cytotoxic mechanisms. It has been proposed that the *N*-terminal domains of Bin and PS2 toxins play a major role in recognizing the target receptors based on their lectin-like structures. Several exposed aromatic residues are located on the surface of the *N*-terminal domain of PS2 and are predicted to interact with the carbohydrate groups of the target receptor on the cancer cell surface [7]. Although some clusters of aromatic residues are present in the *N*-terminal domains of Bin proteins [5], high structural diversity between Bin and PS2 is found in these domains. It was also noted that Bin toxin does not form the SDS-resistant oligomers such as aerolysin protein when exposed to detergents or lipid bilayers [16], indicating the difference between the conformational states of Bin toxin and PS2 when exposed to the plasma membrane of targeted cells. Despite the localization of Oregon Green labeled BinA on the cell membrane of HepG2 cells, the induction of apoptotic events via caspase activation was evident, as shown earlier in Figure 5, indicating the possible interaction of BinA with cell membrane receptors. This characteristic is similar to some other protein toxins such as aerolysin produced by a Gram-negative bacterium *Aeromonas hydrophila* [23], which is reported to cause apoptosis in T cells by forming a small number of channels in the cell membrane, leading to a rapid increase in intracellular calcium levels. As a variety of receptors take part in caspase-dependent apoptosis, identification of the target cell receptors and receptor-binding motifs is expected to provide further insight into the mechanisms of target specificity and cytotoxicity of Bin and PS2 toxins.

In conclusion, we report that the *L. sphaericus* originated Bin toxin, having preferential cytotoxicity towards cancer cells, acts by mechanisms different than those of PS2. Our results suggest that Bin toxin triggers the apoptotic events by caspase induction in HepG2 cells. Altogether, our findings suggest that Bin toxin could be potentially engineered, e.g., by tagging with the tumor-specific peptides, and might serve as an intracellular delivery vector of anticancer therapeutics to target mitochondrial compartments to trigger apoptotic cell death of human cancer cells.

## 3. Materials and Methods

### 3.1. Expression, Purification and Activation of Bin Toxin and Parasporin-2

BinA and BinB proteins were produced as His-tagged proteins from *E. coli* BL21 (DE3) pLysS containing pRSET C-BinA and pET28-BinB, respectively, and purified as described previously [24]. The full-length PS2 gene was de novo synthesized (GenScript, Piscataway, NJ, USA) based on the available protein sequence of PS2 (NCBI accession number AB099515.1) and cloned into pET-28b (+) to express as a His-tagged fusion protein using *E. coli* BL21(DE3) pLysS as a host strain. The expressed PS2 as inclusion bodies were dissolved in 50 mM Na_2_CO_3_ (pH 10.5) and were further purified by HisTrap chelating HP 5 mL column (GE Healthcare Lifesciences, Chicago, IL, USA) pre-charged with Ni^2+^ ions. Those eluted His tagged proteins with imidazole were concentrated by using Amicon Ultra 50 centrifugal filters (Merck, Darmstadt, Germany) with molecular cut-off of 30 kDa for Bin toxin and PS2. The concentrated 6 × (His)-BinA and BinB were then activated by trypsin (0.1 mg/mL) in a 1:20 (*w*/*w*) ratio and 6 × (His)-PS2 was activated by proteinase K (1 mg/mL) in a 1:100 (*w*/*w*) ratio. Activated toxins were separated by size exclusion chromatography using Superdex 200HR 10/300 GL column (GE Healthcare Lifesciences, Chicago, IL, USA) with PBS buffer. After estimation of quality and quantity by SDS-PAGE and Bradford assay, respectively, the purified proteins were filtered with endotoxin-free sterile membrane filters (0.22 µm pore size) and stored at −20 °C.

### 3.2. Cell Line Culture and Cytotoxicity Analyses

Human fibroblast (Hs68) and human epithelial liver carcinoma (HepG2) cell lines were obtained from Dr. Kanokporn Srisucharitpanit and Dr. Tavan Janvilisri, respectively. Cells were cultivated in Dulbecco’s Modified Eagle Medium (DMEM) (Merck, Darmstadt, Germany) supplemented with 10% fetal bovine serum (FBS) and 1% penicillin and streptomycin, maintained at 37 °C in a 5% CO_2_ incubator.

One-dose cytotoxicity assay was carried out by determining the numbers of viable and dead cells using a trypan blue dye exclusion method as described by Morita et al. [25] with some modifications. Briefly, Hs68 and HepG2 cells were grown at about 2 × 10^5^ cells/well in 96-well plates under sterile conditions until approximately 80% confluence. Cells were then treated with 50 µg/mL of BinA and BinB, a combination of BinA and BinB (BinA + BinB, 1:1) and 0.5 µg/mL of PS2, in 100 µL of growth medium, whereas 1× PBS (pH 7.4) was used as a mock control (MC). After 12 h and 24 h of treatment, both attached and floating cells were collected by trypsinization, and aliquots of the cells were mixed with an equal volume of trypan blue dye. Viable cells (the cells excluding dye) and dead cells (those taking up dye) were counted using a hemocytometer, and cell viability was expressed as the percentage of total cell number. Cell morphologies were also observed after toxin treatments under an inverted light microscope (Nikon Eclipse TS100, Melville, NY, USA) with a 10× objective lens at 24 h after inoculation.

For concentration–response study, the cytotoxicity assay was repeated separately for different protein concentrations prepared in 1× PBS. Hs68 and HepG2 cells were grown as mentioned above and treated with increasing concentrations of PS2 (from 0.001 to 0.4 µg/mL) and Bin toxin (from 10 to 60 µg/mL) in 100 µL of FBS-free medium for 24 h. The level of cytotoxicity was evaluated by 3-(4,5-dimethylthiazol-2-yl)-2,5-diphenyltetrazolium bromide (MTT) according to Mosmann [26] with minor modifications. Briefly, the MTT solution (Invitrogen, Carlsbad, California, USA) was added to the treated cells at a final concentration of 0.05 mg/mL and incubated at 37 °C for 4 h in the dark to generate formazan crystals. Finally, 100 μL dimethyl sulfoxide (DMSO) (Merck, Darmstadt, Germany) was added to dissolve the crystals, and the absorbance was measured after 10 min at 570 nm using Multimode Detector DTX 880 ELISA plate reader (Beckman Coulter, Brea, CA, USA). In each experiment, 1× PBS (pH 7.4) was used as a mock control (MC) and the cell survival rate was determined by comparing the absorbance value with that of the control (MC, 100% viability). Experiments were done in triplicate. The concentration–response curve was plotted and 50% effective concentration (EC_50_) values were deduced from log probit analysis.

### 3.3. Cell Assays

The ability of protein toxins to cause plasma membrane damage in cells was determined by lactate dehydrogenase (LDH) leakage using LDH efflux assay. Hs68 and HepG2 cells were seeded on 96-well clear plates at the density of 2 × 10^5^ cells/well and incubated at 37 °C in 5% CO_2_ until approximately 80% confluence. After incubation, the growth medium for HepG2 cell culture was replaced with a fresh medium containing PS2, BinA, BinB and a combination of BinA and BinB (BinA + BinB, 1:1) at EC_50_. Hs68 cells were tested at 0.5 and 50 µg/mL of PS2 and Bin proteins, respectively. Cells were exposed to protein toxins for various time periods. Experiments were performed in triplicate, and in each experiment, 1× PBS (pH 7.4) was used as a solvent control/MC. After the exposure, the LDH efflux from damaged cells was measured in the medium using LDH-Cytotoxicity Assay Kit II (Abcam (ab65393), Cambridge, MA, USA) according to the manufacturer’s protocol. The LDH leakage from the treated cells was compared to the untreated control cells (low control) and cells treated with lysis solution (high control) by measuring the absorbance of reaction mixtures at 450 nm using a microplate reader (Biochrom EZ Read 2000, Cambridge, UK).

Apoptotic detection in cells via Casp3, Casp8 and Casp9 activities in response to protein toxins was done using Caspase 3, Caspase 8 and Caspase 9 Multiplex Activity Assay Kit (Abcam, ab219915) according to the manufacturer’s protocol. Briefly, Hs68 and HepG2 cells were applied to each of the black wall/clear bottom 96-well plates at the density of 2 × 10^5^ cells/well and incubated overnight at 37 °C in 5% CO_2_. PS2, BinA, BinB and a combination of both at EC_50_ were added to the HepG2 cells. Hs68 cells were tested at 0.5 and 50 µg/mL of PS2 and Bin proteins, respectively. Untreated cells were used as a control. After 24 h incubation, fluorogenic indicators for Casp3, Casp8 and Casp9 activity provided in the kit were added and incubated at room temperature for 60 min in the dark. The caspase activities were monitored in a fluorescence microplate reader (TECAN, infinite 200Pro, Grödig Austria) at the specific wavelengths (Casp3: Ex/Em = 535/620 nm, Casp8: Ex/Em = 490/525 nm and Casp9: Ex/Em = 370/450 nm). Caspase activation was evaluated as fold increases by comparing the readings obtained from treated cells with measurements from control cells. Independent experiments were performed in duplicate.

### 3.4. Labeling of Bin Toxin with Fluorescent Dyes

BinA and BinB were labeled with Oregon Green 514-X and Texas Red-X succinimidyl ester dyes (Invitrogen), respectively, to identify the localization of the proteins when used to treat HepG2 cells. One milliliter of 2.5 mg/mL BinA and BinB was incubated with 100 µL of Oregon Green 514-X succinimidyl ester dye and Texas Red-X succinimidyl ester dye (5 mg/mL in DMSO), respectively, at room temperature for 2 h with continuous shaking in the dark. The mixture was applied to a PD-10 column (GE Healthcare, Chicago, IL, USA) to remove unreacted fluorescent reagents that were already equilibrated with PBS buffer pH 7.4. The labeled proteins, namely Oregon Green labeled BinA and Texas Red labeled BinB, were isolated and aliquoted for the experiments.

### 3.5. Bin Toxin Internalization and Localization in the HepG2 Cells

HepG2 cells were used to study the internalization and the localization of the Bin toxin. Cells were seeded on sterilized coverslips placed on a 24-well plate at a density of 1 × 10^5^ cells/well and incubated at 37 °C in a 5% CO_2_ incubator until approximately 80% confluence. Equimolar concentrations of Oregon Green labeled BinA and Texas Red labeled BinB (1:1) were combined and added into the cells at 25 and 50 μg/mL to identify the colocalization pattern of BinA and BinB when internalized into the cells. All treatments were incubated in the dark at 37 °C in a 5% CO_2_ incubator for 2 and 3 h. The treated cells were fixed on the coverslips and prepared for microscopic image analysis. First, the treated solutions were removed and washed thoroughly using 1× PBS buffer pH 7.4 three times. After removing PBS, treated cells were incubated with 400 µL fixative solution (4% paraformaldehyde in PBS, pH 7.4) for 15 min at room temperature, followed by washing with PBS again. The nuclear DNA of cells was stained with 20 ng/µL of DAPI (4’,6-diamidino-2-phenylindole) reagent and incubated for 5 min at room temperature. After staining, the DAPI was removed and washed with PBS. The coverslips with the fixed cells were then placed on a glass slide prior to confocal laser scanning microscopy analysis. The microscopic images were captured with a confocal laser scanning microscope (Zeiss LSM800, Jena, Germany) using Plan-Apochromat 63×/1.4 oil DIC ꝏ/0.17 objective lens. The Oregon Green labeled BinA, Texas Red labeled BinB and DAPI were excited at 488, 559 and 358 nm and exhibited fluorescence emission at 520, 612 and 461 nm, respectively. Quantitative analysis of colocalization was performed using Zen Blue 2.1.57.10000 software. Approximately 30 cells for each condition were analyzed.

### 3.6. Mitochondrial Signal Detection

The association of the internalized signals with the mitochondria in HepG2 cells was studied after treatment with the Bin toxin as mentioned above. The mitochondria were fluorescently stained by using 250 nM of Mitotracker Red CMXRos dye (Invitrogen/Molecular Probes, Eugene, OR, USA) dissolved in anhydrous DMSO followed by washing with 0.1% Tween 20 in PBS. The mitochondria were visualized under a confocal laser scanning microscope (Zeiss LSM800, Jena, Germany) using Plan-Apochromat 63×/1.4 oil DIC ꝏ/0.17 objective lens at excitation wavelength of 579 nm and emission wavelength of 599 nm.

## Figures and Tables

**Table 1:** Cytotoxic activity of protein toxins against HepG2 cells. EC_50_ values were calculated from the data shown in Figure 3 by log probit analysis.

Toxins	EC_50_ (µg/mL)
PS2	0.024
BinA	46.56
BinB	39.72

## Data Availability

The data presented in this study are available within the manuscript.
